# Hybrid morphology dependence of CdTe:CdSe bulk-heterojunction solar cells

**DOI:** 10.1186/1556-276X-9-593

**Published:** 2014-10-29

**Authors:** Furui Tan, Shengchun Qu, Weifeng Zhang, Zhanguo Wang

**Affiliations:** 1 Key Laboratory of Photovoltaic Materials, Department of Physics and Electronics, Henan University, Kaifeng 475004, People’s Republic of China; 2 Key Laboratory of Semiconductor Materials Science, Institute of Semiconductors, Chinese Academy of Sciences, Beijing 100083, People’s Republic of China

**Keywords:** Hybrid bulk-heterojunction solar cells, CdSe, CdTe

## Abstract

A nanocrystal thin-film solar cell operating on an exciton splitting pattern requires a highly efficient separation of electron-hole pairs and transportation of separated charges. A hybrid bulk-heterojunction (HBH) nanostructure providing a large contact area and interpenetrated charge channels is favorable to an inorganic nanocrystal solar cell with high performance. For this freshly appeared structure, here in this work, we have firstly explored the influence of hybrid morphology on the photovoltaic performance of CdTe:CdSe bulk-heterojunction solar cells with variation in CdSe nanoparticle morphology. Quantum dot (QD) or nanotetrapod (NT)-shaped CdSe nanocrystals have been employed together with CdTe NTs to construct different hybrid structures. The solar cells with the two different hybrid active layers show obvious difference in photovoltaic performance. The hybrid structure with densely packed and continuously interpenetrated two phases generates superior morphological and electrical properties for more efficient inorganic bulk-heterojunction solar cells, which could be readily realized in the NTs:QDs hybrid. This proved strategy is applicable and promising in designing other highly efficient inorganic hybrid solar cells.

## Background

Solar cells based on nanoparticles have attracted intense attention in view of their compatibility with the solution synthesis of materials, low-cost fabrication of devices, and large area flexibility. Compared with their counterparts of organic solar cells which also possess these potentials, nanocrystal thin-film solar cells offer easy tuning of light response in a broad range by tuning the quantum size effect of colloidal nanoparticles. Up to now, tremendous attention has been paid to photovoltaic nanomaterials which could be adopted in thin-film solar cells, such as PbS [1-4], CuInS_2 _[5-7], and CdTe [8,9].

With regard to the presently researched photovoltaic device with Schottky contact or bilayer heterojunction structure, it was suggested that the photocurrent was generated from charge separation driven by the built-in electric field in the depletion region which is located at the semiconductor-metal contact [10,11] or p-n interface [12,13]. Photogenerated excitons must diffuse a long way to the p-n depletion region before their splitting, which takes a high risk of recombination considering a relatively large quantum dot (QD) thickness as well as a small depletion width. To resolve this problem, a hybrid bulk-heterojunction (HBH) nanostructure was adopted [14], as what is commonly used in organic thin-film solar cells that possess highly efficient exciton splitting and charge transfer properties. Another critical problem adopting this structure in inorganic solar cell is that continuous charge transportation should also be required at the same time. Thus, it might not be a good idea if both spherical-shaped p- and n-type nanocrystals are blended together, which makes it difficult to form network pathways for electrons and holes. Nanocrystals with a hyperbranched shape should overcome the trade-off between efficient exciton separation and charge transportation and collection. Thus, in our previous work, the HBH concept was successfully introduced to fabricate all-inorganic QD solar cells based on CdTe nanotetrapods (NTs) and CdSe QDs, which can form a HBH nanostructure with a type-II energy band alignment [15].

It may be noticed that the morphology of inorganic nanoparticles has a profound impact on the performance of organic/inorganic hybrid solar cells [16,17]. Similar to all-inorganic solar cells that also employ a hybrid bulk-heterojunction structure, how the hybrid morphology affects their properties should be explored, which, however, has not been reported on this new device. Here in this work, a focused investigation is carried out on the hybrid structure dependence of photovoltaic performance through variation in CdSe nanoparticle morphology while keeping the CdTe NTs unchanged. QD- or NT-shaped CdSe was introduced as an electron acceptor and transporter in the CdTe:CdSe hybrid. The hybrid structure in a nanoscale of the thin film is found to be closely related with the morphology of CdSe nanoparticles. Correspondingly, the charge dynamics behavior at the interface shows obvious difference in the two hybrids, which further results in variation in the photovoltaic performance of the two solar cells.

## Methods

### Synthesis of CdTe and CdSe nanoparticles

CdTe NTs and CdSe QDs were synthesized according to the procedure in the literature [18] with some modifications. A Cd precursor solution (containing 1 mmol of CdO dissolved in 3 ml of oleic acid and 3 g tri-*n*-octylphosphine oxide (TOPO)) was heated to 140°C and kept at this temperature for 1 h under nitrogen protection. In another flask, a Te source solution was formed by dissolving 0.5 mmol Te powder in 3 ml tri-*n*-octylphosphine (TOP). The Cd stock solution was heated to 260°C, and then the Te solution was quickly injected. The reaction proceeded for 3 to 4 min at 260°C to produce CdTe nanocrystals with a tetrapod shape. As to the CdSe QDs, a similar recipe and procedure were used just by replacing Te with 1.0 mmol Se powder. CdSe NTs were synthesized according to the procedure in the literature [19]. CdO (1 mmol), oleic acid (OA, 6 mmol), and 20 ml 1-octadecene (ODE) were pumped at 140°C under N_2_ flow for 30 min. After that, the temperature was firstly raised to about 240°C where the solution turned clear and then decreased to 190°C at which a TOP-Se-hexadecyltrimethylammonium bromide (CTAB) solution (containing 1 ml TOP, 0.5 mmol Se, 0.05 mmol CTAB, and 3 ml toluene) was injected quickly. The injection caused the temperature to drop to about 165°C where the reaction was allowed and persisted for an hour to grow the CdSe NTs. Then, the heating mantle was removed and the solution was cooled to room temperature, after which 10 ml acetone was injected to collect the red precipitation by centrifugation at 4,500 rpm. All the three nanoparticles were purified with chlorobenzene/ethanol solvent/antisolvent for at least six times. The final products were dissolved separately in chlorobenzene to form solutions with desired concentration.

### Fabrication of hybrid solar cells

The hybrid bulk-heterojunction solar cell was fabricated as follows: firstly, patterned indium tin oxide (ITO)-coated glass substrates were cleaned sequentially with soap water, deionized water, acetone, and isopropanol under ultrasonication for 20 min. Substrates were then dried under N_2_ flow, after which a compact TiO_2_ layer was deposited on top by spin coating a titanium-acetylacetone precursor and then sintering at 450°C for 90 min. The active layer was produced by spin coating several layers of CdTe NTs:CdSe QDs or CdTe NTs:CdSe NTs hybrid. The *w*/*w* ratio of CdTe to CdSe was varied in the hybrid. Following each spin coating, the substrates were treated with solvent containing 3-mercaptopropionic acid (MPA)/methanol solution (10% by volume). For solvent treatment, two drops of MPA/methanol solution were dispensed onto the CdTe layer or CdTe:CdSe hybrid layer, and the substrate was spun at 2,500 rpm for 15 s after a 6-s wait. Three rinse steps with methanol were applied under the same operation. Afterward, the substrates were annealed at 150°C for 10 min. The solar cell fabrication was finished by thermally depositing 5 nm MoO_3_ and thereafter 150 nm Au on top.

### Characterization

The morphology of CdTe and CdSe nanoparticles was confirmed by transmission electron microscopy (TEM) on a Hitachi H-800 (Hitachi, Ltd., Tokyo, Japan) at an acceleration voltage of 80 kV. The HBH thin-film surface and cross-sectional morphology were measured by field-emission scanning electron microscopy (FESEM, JEOL 7006 F, JEOL Ltd., Tokyo, Japan). Atomic force microscopy (AFM) test was carried out on a Solver P47 SPM (NT-MDT Co*.*, Eindhoven, The Netherlands) under semi-contact mode. Absorption measurements were carried out on a Varian Cary ultraviolet-visible-infrared spectrophotometer (5000 model, Varian Inc., Palo Alto, CA, USA). Electrochemical impedance spectra were recorded on a CHI 660E electrochemical workstation (Chenhua Instruments, Inc., Shanghai, China). The current-voltage (*I*-*V*) measurements on CdTe:CdSe HBH solar cells were performed on a Keithley 2400 source in forward bias mode (Keithley Instruments Inc., Cleveland, OH, USA) under air mass (AM) 1.5 (100 mW/cm^2^) illumination.

## Results and discussion

**Figure 1:**
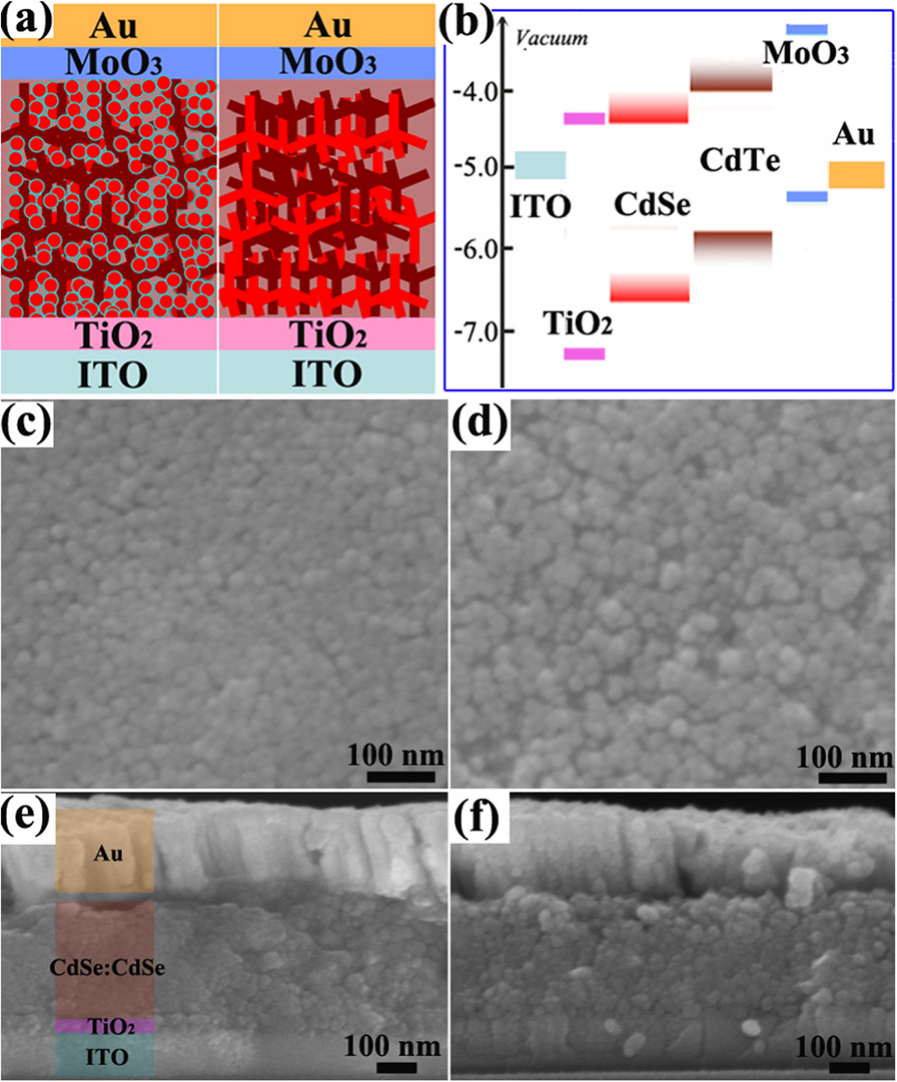
**Solar cell structure and hybrid film morphology. (a)** The skeleton of the two solar cells. **(b)** Energy level alignment of the entire solar cell. **(c)** SEM topology of NTs:QDs solar cell. **(d)** SEM topology of NTs:NTs solar cell. The cross-sectional SEM images of the **(e)** NTs:QDs solar cell and **(f)** NTs:NTs solar cell.

The skeleton of the two solar cells is firstly given in Figure 1a with the following structure: ITO/TiO_2_/CdTe:CdSe/MoO_3_/Au. The optimized thickness for the TiO_2_ and MoO_3_ buffer layers are 40 and 5 nm, respectively. The energy level alignment of the solar cells is shown in Figure 1b where a type-II heterojunction at the CdTe:CdSe interface enables efficient exciton dissociation and charge transfer. The energy level alignment of CdTe and CdSe was selected according to ref. [8]. Hybrid thin films composed of NTs:QDs or NTs:NTs exhibit different surface features. The NTs:QDs film looks more uniform with a flat and crack-free surface (Figure 1c), while the NTs:NTs film has more pinholes and large particle aggregation (Figure 1d). With the CdSe QDs infiltrating into the network of hyperbranched CdTe, the NTs:QDs hybrid seems more favorable for the formation of a densely packed film than the NTs:NTs hybrid in which CdTe NTs and CdSe NTs are interpenetrated to form networks. This also could be reflected from the cross-sectional view of the two solar cells in Figure 1e,f. Note that the optimized thickness of the hybrid layer for NTs:QDs and NTs:NTs solar cells is similar, about 280 nm.

**Figure 2:**
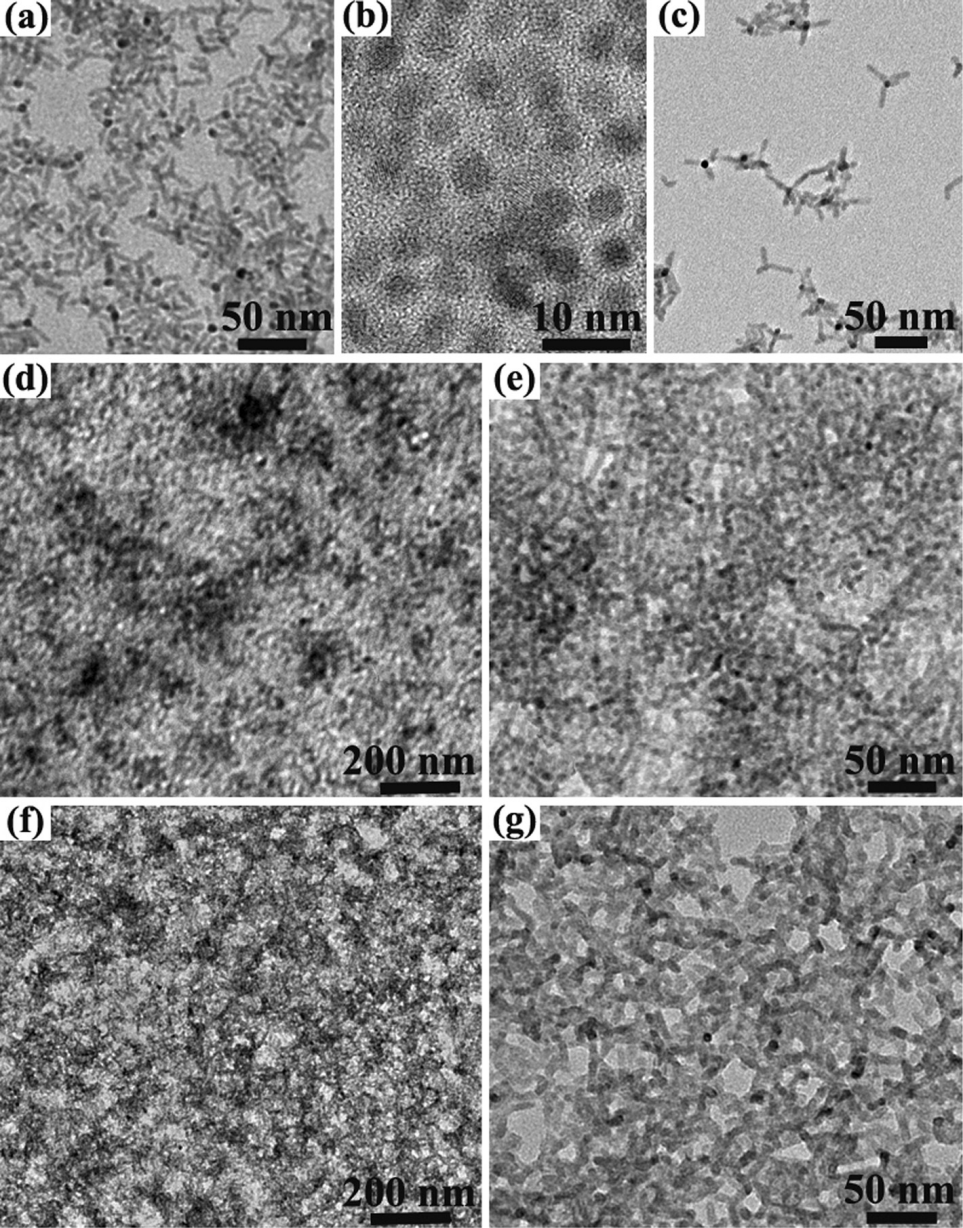
**Morphology of the three kinds of nanoparticles and their hybrid in the thin film. (a)** CdTe NTs. **(b)** CdSe QDs. **(c)** CdSe NTs. **(d)** CdTe:CdSe QDs-2:1 hybrid. **(e)** Enlarged view of **(d)**. **(f)** CdTe:CdSe NTs-3:1 hybrid. **(g)** Enlarged view of **(f)**.

To deeply observe the detailed morphology of particle aggregation in the film, we prepared an ultrathin film that could be clearly detected by TEM. The composed CdTe NTs, CdSe QDs, and NTs are firstly given in Figure 2a,b,c, respectively. All the three nanoparticles can be dispersed well in an organic solvent, i.e., chlorobenzene and chloroform, which ensures a uniform phase separation in the hybrid. However, the packing and aggregation of these nanobuilding blocks are clearly different in the two hybrid thin films. Homogeneous packing of nanoparticles with high density is demonstrated in the NTs:QDs hybrid film (Figure 2d). The densely aggregated and closely interconnected nanocrystals can be observed (Figure 2e), which is ascribed to the infiltration of QDs into the network of NTs. In comparison, the NT network with large amounts of pinholes is displayed in the NT hybrid (Figure 2f,g). Although CdTe and CdSe NTs cannot be exactly identified in the hybrid, their loose distribution and attachment are clearly evident. The film quality in the microregion, or in other words, the nanoparticle assembly and aggregation, will affect its electrical behaviors such as charge transfer at the nanoparticle interfaces, their collection and recombination, or current leaking [20].

**Figure 3:**
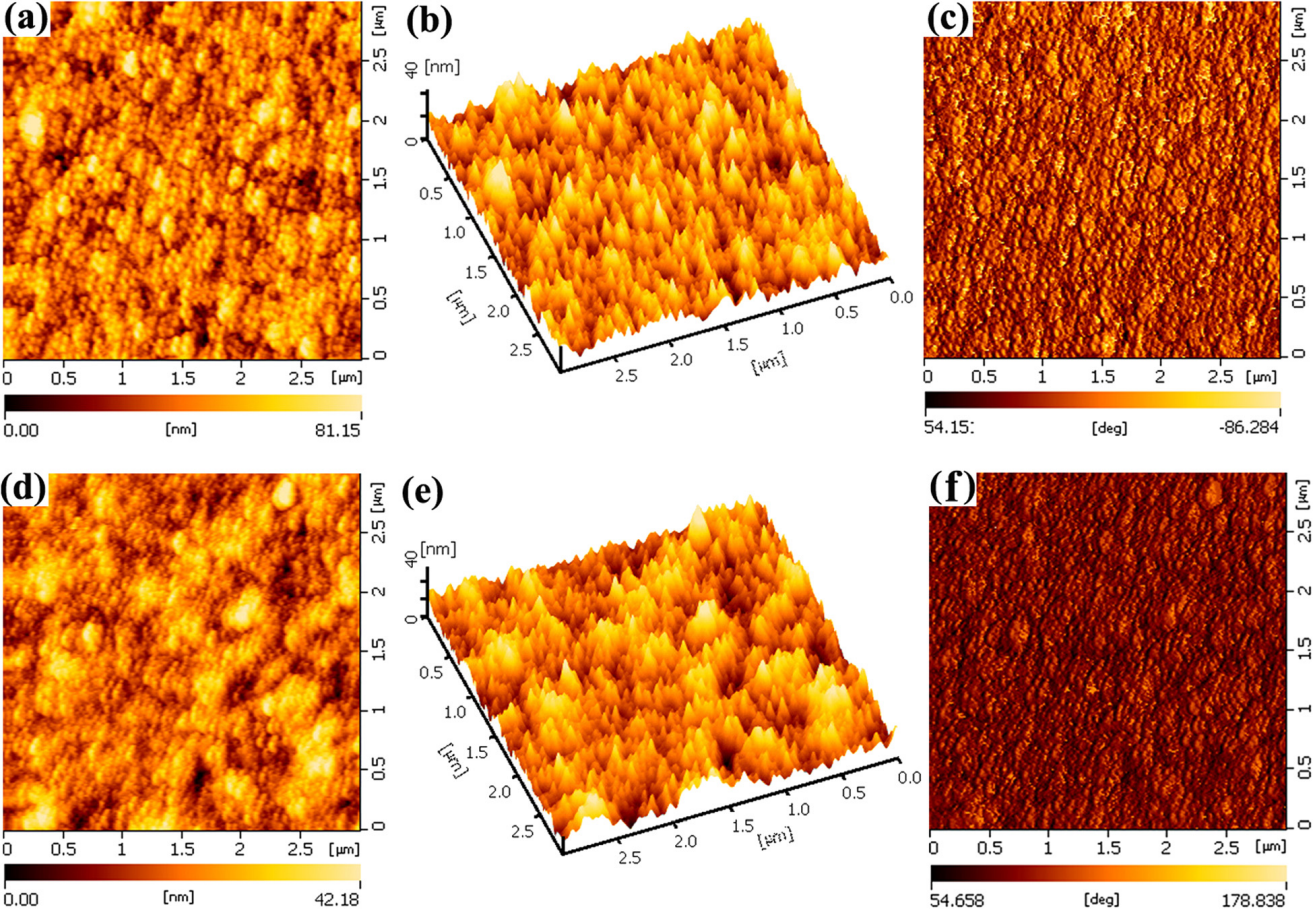
**AFM images of the two hybrid films.** Height and phase images of the **(a-c)** NTs:QDs hybrid thin film and **(d-f)** NTs:NTs hybrid thin film.

The hybrid film topography was studied using AFM, as shown in Figure 3. A smooth and uniform surface is exhibited on the NTs:QDs hybrid film, with a root-mean-square (RMS) roughness of 4.9 nm. Ordered and regular particle arrangement can be observed, and the surface phase is also very uniform. Compared to the NTs:QDs hybrid, the interpenetration of CdTe and CdSe NTs seems to prevent their dense packing in the hybrid film. Nanoparticle regular distribution in the NTs:NTs hybrid film is slightly affected due to the large-sized aggregation. Thus, the RMS is slightly increased to about 6.7 nm. Still, the surface phase separation is small and uniform. The flat surface of the two hybrid films enables a good electronic contact between the hybrid film and the above MoO_3_ buffer layer.

**Figure 4:**
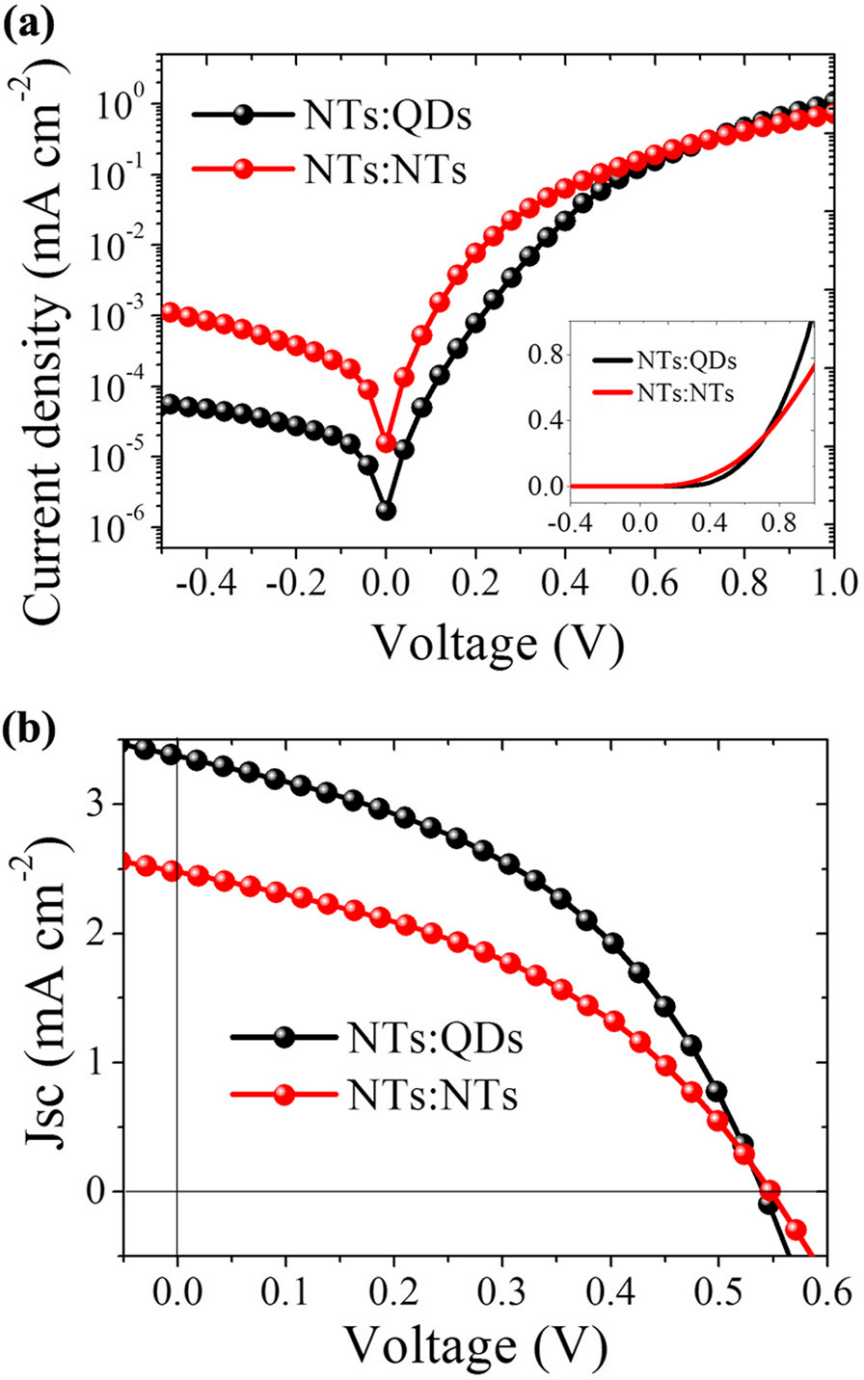
**Photovoltaic performance of the solar cells.***I*-*V* characteristics of the two solar cells **(a)** in the dark and **(b)** under 100 mW cm^-2^ light illumination.

In order to evaluate the electrical properties of the two hybrid structures, the *I*-*V* characteristics of the two solar cells were measured in the dark, and the results are shown in Figure 4a. As can be seen from the figure, the reverse current in the NTs:QDs hybrid device is smaller than that in the NTs:NTs solar cell, which means a smaller current leakage in the former hybrid structure than in the latter. A decreased leakage current reflects a confined reverse transfer of charges [21]. As the hybrid heterojunction for the two structures is constructed both with CdTe and CdSe, the difference in charge transfer property at the interface is thus attributed to the electrical contact caused by different hybrid morphologies as shown in Figure 2d,e,f,g, that is, the densely packed NTs:QDs hybrid is more effective in suppressing reverse transfer of electrons from CdSe to CdTe or/and from cathode to anode. Under AM 1.5 (100 mW cm^-2^) illumination, the NTs:QDs hybrid solar cell generates a photocurrent density (*J*_sc_) of 3.4 mA cm^-2^, open circuit voltage (*V*_oc_) of 0.54 V, fill factor (FF) of 44%, and optic-electric conversion efficiency (Eff) of 0.8%, which is much superior than the NTs:NTs hybrid device with a *J*_sc_ of 2.5 mA cm^-2^, *V*_oc_ of 0.54 V, FF of 41%, and Eff of 0.56%. The obvious increase in *J*_sc_ dominates the performance improvement in the NTs:QDs hybrid solar cell.

**Figure 5:**
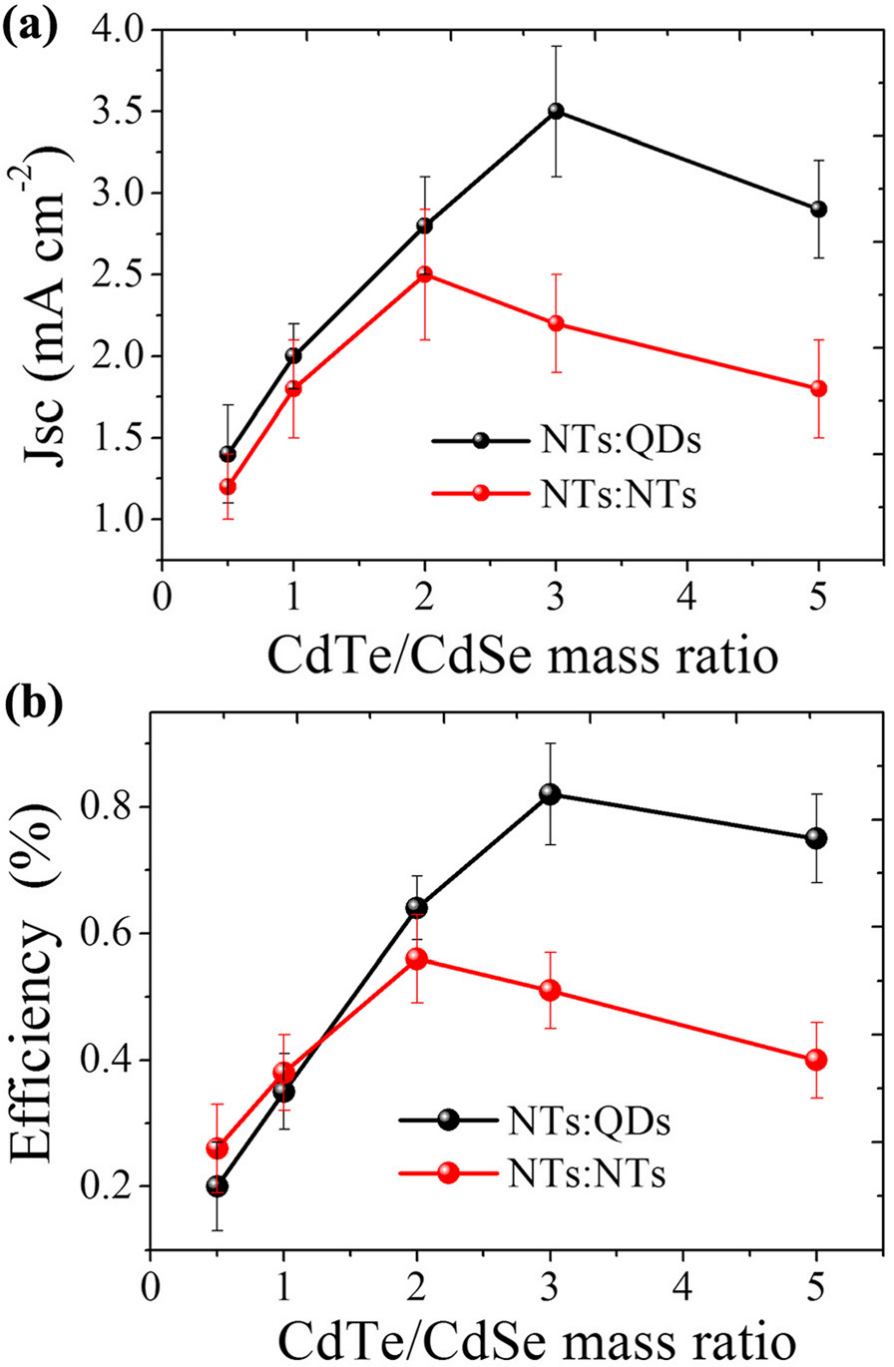
**CdTe:CdSe mass ratio dependence of photovoltaic performance. (a)***J*_sc_ and **(b)** efficiency.

**Figure 6:**
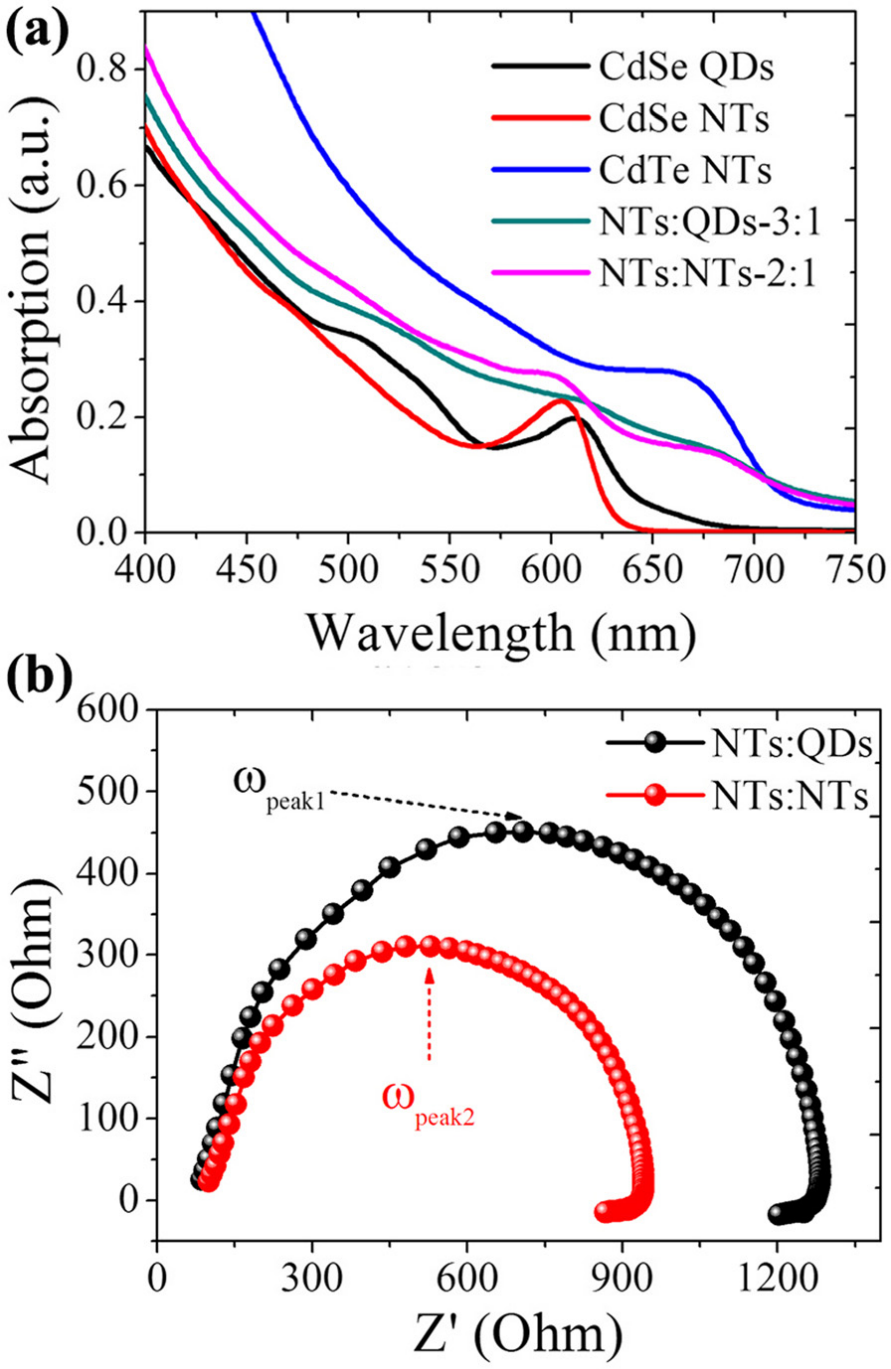
**Light absorption and electrochemical impedance of the two solar cells. (a)** Light absorption properties of the solar cells with optimized active layers. **(b)** Electrochemical impedance spectra of the two cells. *ω*_peak_ in **(b)** means peak frequency.

The performance of inorganic hybrid solar cells was found to be greatly dependent on the phase separation of the two materials which is controlled by their mass ratio. As shown in Figure 5a, *J*_sc_ values of NTs:QDs and NTs:NTs hybrid solar cells exhibit a similar tendency following the variation in CdTe/CdSe mass ratio. It reaches the maximum at a mass ratio of 3:1 in the NTs:QDs hybrid solar cell and 2:1 in the NTs:NTs cell. The observed variation in *J*_sc_ is probably caused by changes in phase separation which greatly influences charge transfer at the interface and collection through a separate charge channel. The mass ratio dependence of conversion efficiency, as shown in Figure 5b, is of great similarity with the tendency of the *J*_sc_ values. The mass ratio at the efficiency maximum, like that in the *J*_sc_ data, is larger in the NTs:QDs hybrid than in the NTs:NTs hybrid, meaning that a relatively small mass ratio of CdSe QDs is needed to form an optimized hybrid nanostructure for solar cells. By infiltrating the interspaces of branched CdTe NTs, The CdSe QDs are more effective in constructing interpenetrated hybrid networks for efficient transfer and transport of charges. It is noticed that the photovoltaic performance of the NTs:QDs solar cell is worse than that of the NTs:NTs hybrid at a mass ratio smaller than 1, probably because of the relatively isolated CdTe NT phase surrounded by the larger amount of CdSe QDs in comparison with CdSe NTs. This also demonstrates that a well-worked solar cell strongly requires a suitable phase separation that is good to balanced charge transportation.To further validate the reason of performance difference, the light absorption properties of the two solar cells are compared in Figure 6a. Also given are the light absorption properties of dispersed CdSe and CdTe nanoparticles in chlorobenzene. The CdTe NTs and CdSe NTs or QDs show distinct exciton absorption near 675 and 610 nm, respectively. Both the NTs:QDs and the NTs:NTs hybrid films show a superposition of light absorption of CdTe and CdSe. A slightly increased intensity at the short wavelength is observed for the NTs:NTs hybrid solar cell compared to the NTs:QDs cell, due to the comparatively larger CdSe NT content in the optimized active layer. The characteristic exciton peak of CdSe nanoparticles at about 610 nm is thus more apparent for NTs than for QDs. From the absorption comparison here, one can say that some other aspects (such as exciton dissociation or charge collection), not the light absorption, should be responsible for the performance superiority of the NTs:QDs hybrid solar cell.

**Figure 7:**
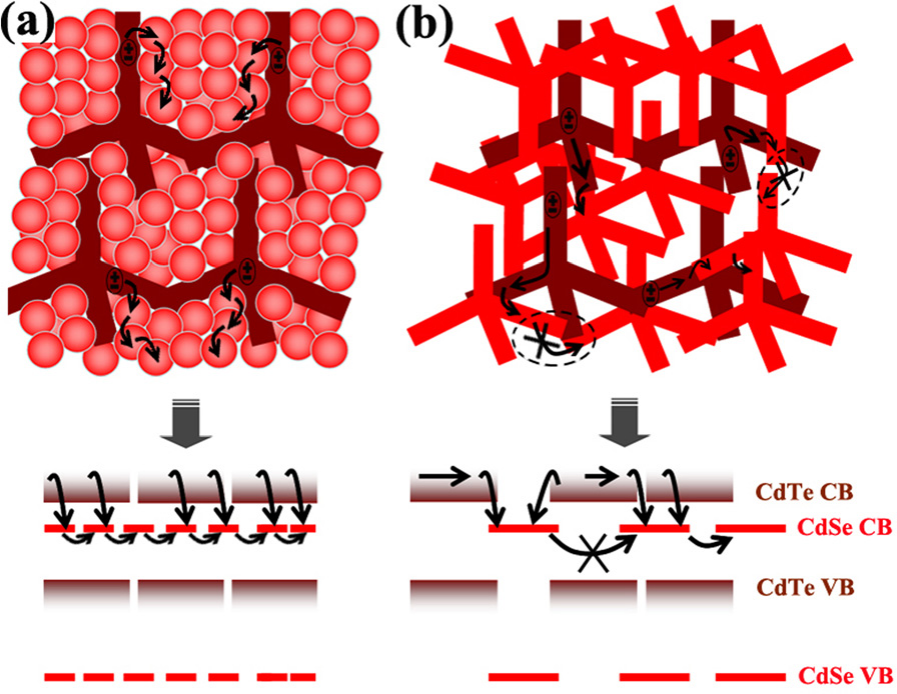
**Skeleton of hybrid phase and charge transfer.** Nanoscale hybrid morphology and electrons transfer and transport in **(a)** CdTe NTs:CdSe QDs blend and **(b)** CdTe NTs:CdSe NTs blend.

The influence of hybrid structure on charge transfer and transport in the hybrid is researched using electrochemical impedance spectrum (EIS), and the results are shown in Figure 6b. In the measurement, mainly one semicircle was observed on the Nyquist plot for each cell under an applied voltage of 0.5 V. The arc in each cell corresponds to the charge transfer and thereafter transportation process because of its main response in the middle (100 to 10 KHz) and low frequency range (10 KHz to 1 Hz) [22-24]. The recombination resistance (*R*_ct_), indicated from the diameter of the semicircle, is enlarged in the NTs:QDs hybrid solar cell compared to that in the NTs:NTs cell. The *R*_ct_ values obtained here represent charge recombination that happens not only at the electron donor-acceptor interface but also during their jump-tunneling to the electrodes. It demonstrates that the recombination process is suppressed in the NTs:QDs hybrid compared to the NTs:NTs structure. Besides, the peak frequency (*ω*_peak_), as a representation of the effective recombination rate (*k*_eff_) [22], is found to be smaller in the NTs:QDs hybrid (*ω*_peak1_ = 10,240 Hz) than in the NTs:NTs hybrid (*ω*_peak2_ = 14,785 Hz), also indicating a decreased charge loss in the recombination process in the former structure. The electron lifetime, obtained from *τ* =1/2π*ω*_peak _[25], is calculated to be 15.6 μs in the NTs:QDs hybrid and 10.7 μs in the NTs:NTs hybrid. Thus, the NTs:QDs heterojunction is also beneficial to charge collection due to the slightly increased lifetime in the hybrid film.Considering the detailed arrangement of CdTe and CdSe nanoparticles in the nanoscale of the hybrid film, we give a speculative reason for the above difference of charge dynamics. As seen in Figure 7a, dense and ordered assembly of CdSe QDs is readily formed in the network of branched CdTe, which enables a close heterojunction contact with a large interface area. Thus, photogenerated excitons in CdTe are easily split at the interface all around, without a long-ranged diffusion. Transport of electrons among CdSe QDs is also convenient because of the densely package of QDs. In comparison, as the loosely entangling and crossing contact between CdTe and CdSe NT arms is comparatively small in the contact area, some excitons have to diffuse a long way until their splitting at the interface, so that the dissociation efficiency is negatively influenced. Besides, continuous transport of electrons among CdSe NTs would be confined and interrupted at times if the interconnection of CdSe NTs is destroyed by the many surrounding CdTe NTs, a thick potential barrier inhibiting electron transfer. Therefore, charge recombination is comparatively high in the NTs:NTs hybrid, which could affect the electron lifetime, as depicted in EIS characterization in Figure 6.

## Conclusions

In conclusion, we have researched the influence of hybrid morphology on the photovoltaic performance of inorganic bulk-heterojunction solar cells. CdSe QDs or NTs were adopted together with CdTe NTs to form the hybrids that behave differently in film topology as well as charge transfer and transport. Compared to the CdTe NTs:CdSe NTs hybrid, interpercolation of CdTe NTs and CdSe QDs enables a flat and densely packed hybrid film, which ensures a better electrical contact between the hybrid active layer and the anode buffer layer. Besides, an interpenetrated heterojunction of NTs:QDs with large interface area facilitates electron transfer from CdTe NTs to the closely surrounding CdSe QDs, and electron transport also benefits from the dense and ordered assembly of QDs in the network. The structural and electrical advantage of the NTs:QDs hybrid makes it superior to the NTs:NTs hybrid in optic-electric conversion. Our research provides a designing strategy that should be considered for highly efficient inorganic hybrid bulk-heterojunction solar cells.

## Competing interests

The authors declare that they have no competing interests.

## Authors’ contributions

FRT carried out the experiments and drafted the manuscript. SCQ participated in the sequence alignment. WFZ conceived the study and participated in its design. ZGW participated in the design of the study and performed the analysis. All authors read and approved the final manuscript.
